# Hospitalizations Among Active Component Members of the U.S. Armed Forces, 2023

**Published:** 2024-06-20

**Authors:** Armed Forces Health Surveillance Division Defense Health Agency, US

## Abstract

**What are the new findings?:**

The hospitalization rate among U.S. active component service members in 2023 at both military and non-military medical facilities was 48.8 per 1,000 person-years, approximately 9% lower than the 2022 rate. As in prior years, over half (56.7%) of hospitalizations for active component members were associated with primary diagnoses in 2 categories: mental health disorders and pregnancy conditions. In 2023 COVID-19 accounted for less than 0.1% of total active component hospitalizations, a greater than 85% decline from 0.4% in 2022, and a nearly 96% decline from 1.5% in 2021.

**What is the impact on readiness and force health protection?:**

As in prior years, mental health disorders, including substance abuse disorders, were associated with the longest median hospital stay, 6 days; 5% of hospitalizations for mental health disorders had durations greater than 30 days. Prolonged hospitalizations, after care, and early attrition due to these common disorders can diminish not merely individual but unit operational readiness.

## 1 BACKGROUND

This report documents the frequencies, rates, trends, and distributions of hospitalization among active component members of the U.S. Army, Navy, Air Force, Space Force, and Marine Corps during calendar year 2023. Summaries are based on standardized hospitalization records at U.S. military and non-military (reimbursed through the Military Health System) medical facilities worldwide that are routinely maintained in the Defense Medical Surveillance System (DMSS).

In this report, primary (first-listed) discharge diagnoses are considered indicative of the primary cause of hospitalization. As in prior *MSMR* reports, summaries are based on the first 3 digits of the International Classification of Diseases, 10th Revision (ICD-10) codes of the primary discharge diagnoses. Hospitalizations not routinely documented by standardized, automated records, e.g., during field training exercises or while shipboard, are not available in a centralized location for health surveillance purposes and are excluded from this report. Incidence rates were calculated per 1,000 person-years (p-yrs). Percent change in incidence was calculated using unrounded rates.

**Frequencies, Rates, and Trends**

In 2023, 62,806 hospitalizations were recorded for the active component members of the U.S. Army, Navy, Air Force, Space Force, and Marine Corps (**Table 1**); 45.3% of these hospitalizations were in non-military facilities (data not shown), compared to 46.4% in 2022.

**Figure 1:**
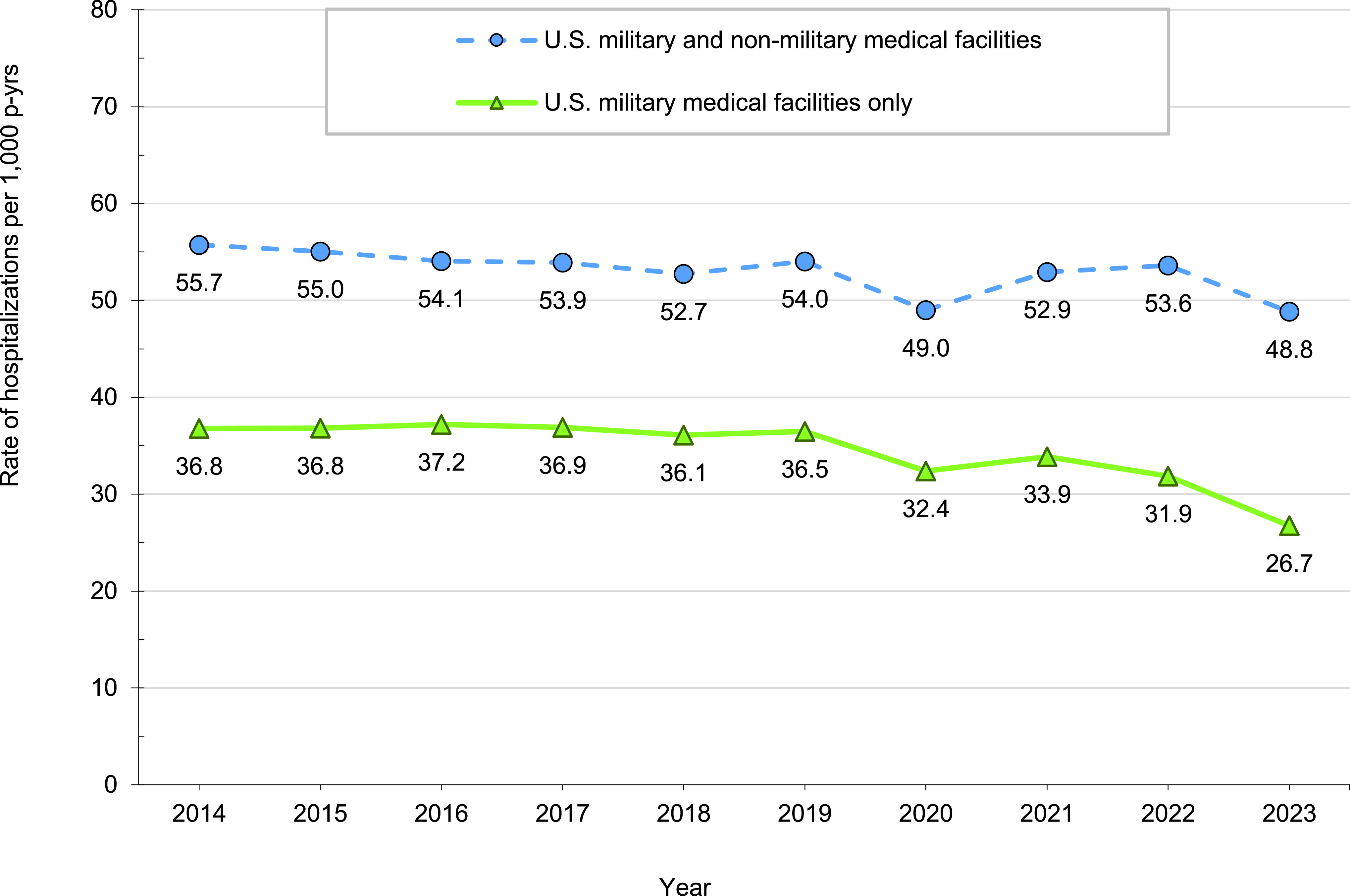
Rates of Hospitalization by Type of Medical Facility, Active Component, U.S. Armed Forces, 2014-2023

Between 2014 and 2023, hospitalization rates manifested a general downward trend, but with annual fluctuations. Prior to 2020, the rates per 1,000 p-yrs fluctuated within a range, from a high of 55.7 in 2014 to a low of 52.7 in 2018, with an average annual percent change of less than 3% (-0.3% to +2.5%). In 2020, however, the hospitalization rate dropped sharply, below the typical range to 49.0, with a 9.4% decrease compared to 2019, the previous year. The rates in 2021 and 2022 then rebounded to their pre-pandemic range. As of April 2024, the crude annual hospitalization rate for 2023 was 48.8 per 1,000 p-yrs, approximately 9.0% lower than the rate in 2022 and comparable to the rate observed in 2020 (**Figure 1**).

**Hospitalizations, by ICD-10 Major Diagnostic Categories**

In 2023, only 4 ICD-10 major diagnostic categories accounted for almost three-quarters (73.0%) of all active component hospitalizations: mental health disorders (31.1%), pregnancy- and delivery-related conditions (25.6%), injury and poisoning (8.7%), and digestive system disorders (7.6%) (**Table 1**). Consistent with the findings in 2019 and 2021, hospitalizations for mental health disorders in 2023 accounted for more than any other major diagnostic category; 2009 was the last year in which another diagnostic category, pregnancy- and delivery-related conditions, surpassed hospitalizations for mental health disorders (data not shown). COVID-19 accounted for less than 0.1% of total hospitalizations among active component service members (ACSMs) in 2023, a decline greater than 85% from the previous year, when 0.4% of hospitalizations were due to COVID-19, and represents a decline of nearly 96% from the 2021 figure of 1.5%.

The latest data indicate that, from 2019 through 2023, both the numbers and rates of hospitalizations decreased for all major diagnostic categories (**Table 1**). The 3 largest declines, by number and percent rate of hospitalization, were observed for musculoskeletal system and connective tissue (1,273 fewer hospitalizations; 24.2% rate decrease) conditions, injury and poisoning (-1,229; -16.6%), and ‘other’ (-1,123; -48.4%). COVID-19 ranked next in terms of decline in hospitalization numbers, at 980, but demonstrated the highest percent decline (-91.9%) in the hospitalization rate. Additional categories with substantially decreased hospital admission rates included respiratory system (-797; -35.0%), signs, symptoms and ill-defined conditions (-860; -25.8%), and skin and subcutaneous tissue (-338; - 29.0%).

The relative proportion of hospitalizations by major diagnostic category was generally stable over the surveillance period (**Table 1**). COVID-19, which was included as a separate diagnostic category in 2020 and ranked thirteenth in total hospitalizations in 2021, dropped to the lowest ranking, eighteenth, in 2023.

**Hospitalizations, by Sex**

In 2023, the hospitalization rate (for all causes) among service women was more than 3 times that of service men (116.5 per 1,000 p-yrs and 34.3 per 1,000 p-yrs, respectively). These data are consistent with national hospitalization rate trends published in 2022 for women and men ages 18-44 years (95 per 1,000 p-yrs and 37 per 1,000 p-yrs respectively) in the general U.S. population.^1^ Excluding pregnancy- and delivery-related conditions, the rate of hospitalizations among women (45.6 per 1,000 p-yrs) was 33.0% higher than among men (34.3 per 1,000 p-yrs) in 2023 (data not shown). This rate difference was primarily due to hospitalizations for mental health disorders (female:male rate difference [RD]: 6.1 per 1,000 p-yrs) and genitourinary disorders (RD: 2.6 per 1,000 p-yrs) (data not shown). Excluding pregnancy- and delivery-related conditions, hospitalization rates were relatively similar among men and women for the remaining 16 major diagnostic categories (data not shown).

**Figure 2:**
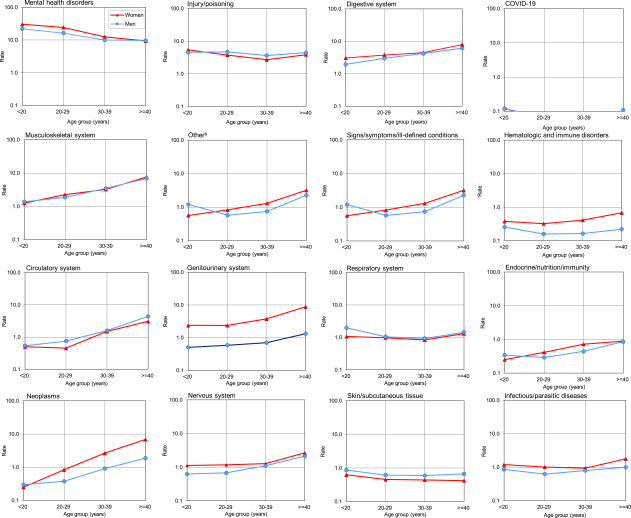
Rates of Hospitalization by ICD-10 Major Diagnostic Category, Age Group, and Sex, Active Component, U.S. Armed Forces, 2023

Relationships between age and hospitalization rates varied by major diagnostic category (**Figure 2**). Rates among women in all age groups were consistently higher for the genitourinary, nervous and digestive system, hematologic and immune disorder, and infectious and parasitic disease categories. The gender gap was greatest for the genitourinary system category and widened with age, with the female-to-male ratio increasing from 4.0 in age categories under age 30 years to 6.6 in those older than 30. Similarly, hematologic and immune disorder rates were higher among women and with age increased from 1.5 to 3.1 times higher in women than men. Additionally, women had progressively higher (from 2.2 to 3.7 times) hospitalization rates within the neoplasms category, except for the youngest age group. In contrast, rates among men were higher than women in all age groups for the skin and subcutaneous tissue as well as respiratory and circulatory system categories. Hospitalization rates of mental health disorders were more than twice as high among younger women, under the 30 years of age, and were comparable among older age groups.

Hospitalization rates among both sexes generally increased with age for most diagnostic categories except mental health, injury and poisoning, skin and subcutaneous tissue, respiratory, and infectious and parasitic diseases. Rates decreased for both sexes with increasing age for mental health disorders and were relatively stable among all age groups for injury, infectious/parasitic diseases, respiratory system disorders, skin and subcutaneous tissue categories, as well as COVID-19.

**Most Frequent Diagnoses**

Mental health disorders represented a significant portion of hospital admissions among ACSMs. Adjustment disorders were the primary discharge diagnosis among both men (n=4,861) and women (n=1,321) (**Tables 2** and **3**) in 2023, accounting for nearly 10% of total hospitalizations. The next 4 most frequent diagnoses, for both sexes, were alcohol- and depression-related disorders, including recurrent major depressive disorder (severe without psychotic features), and post-traumatic stress disorder (PTSD). Mental health disorder diagnoses, collectively, accounted for over 40% of all hospitalizations among men and, excluding pregnancy- and delivery-related conditions, among women.

Pregnancy- and delivery-related conditions constituted the top major diagnostic category for women, accounting for over three-fifths (60.8%) of all female hospitalizations, although adjustment disorders represented the most frequent cause of hospitalization when examining ICD-10 diagnoses through the fourth character code (**Table 3**).

Other common causes of hospitalization, regardless of sex, included ‘other and unspecified acute appendicitis’, ‘sepsis, unspecified organism’, and ‘other symptoms and signs involving emotional state’, as well as ‘other specified disorders of muscle’ for men, and ‘abnormal uterine and vaginal bleeding’ for women.

**Hospitalization Durations**

**Figure 3:**
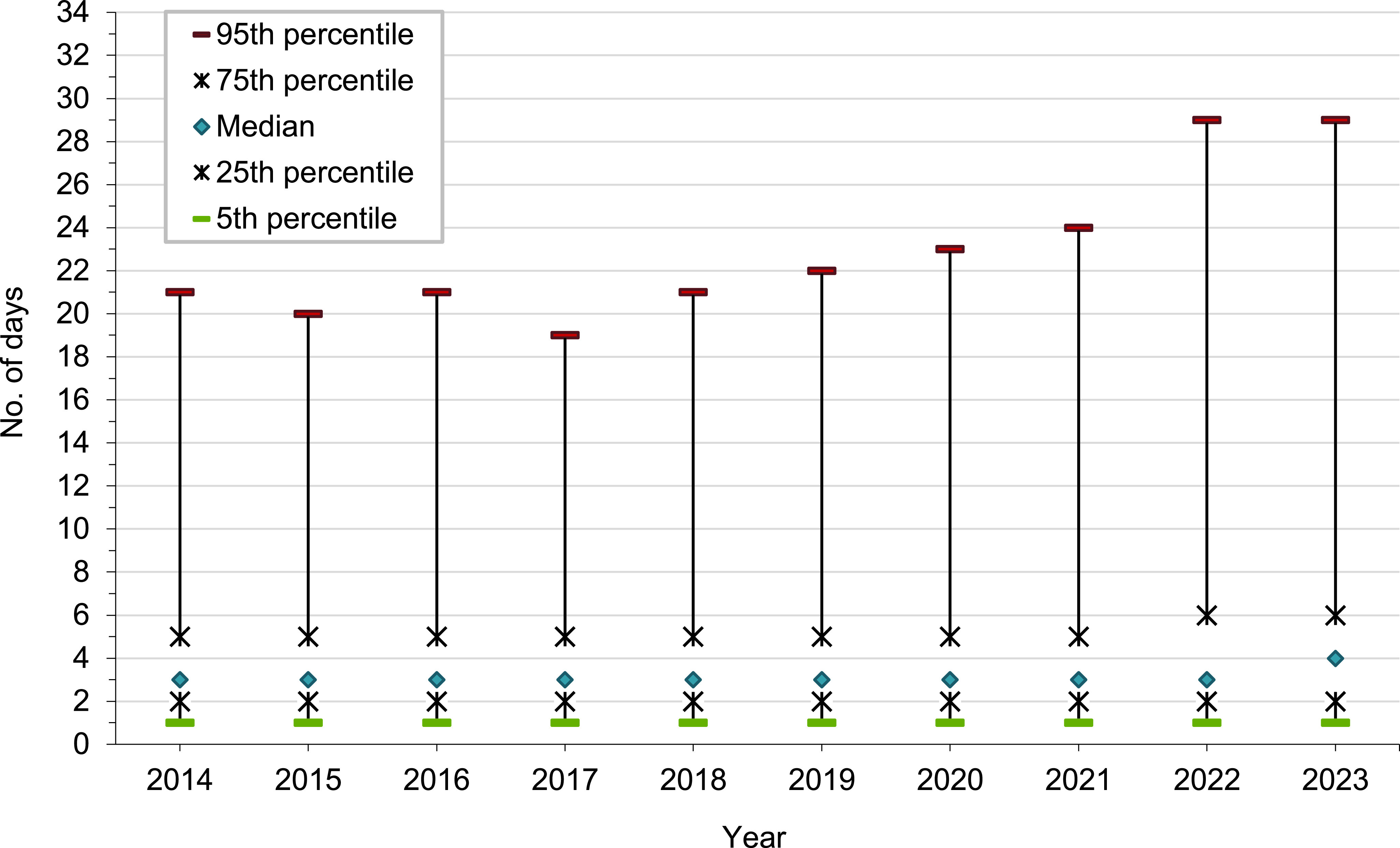
Length of Hospital Stay, Active Component, U.S. Armed Forces, 2014-2023

When graphically represented, hospitalization durations demonstrate a highly right-skewed (positive) distribution, with the lower limit equal to 1 day and a mode of 3 days. Because length of hospital stay is not normally distributed, the median duration with interquartile range (IQR) was chosen as the best measure of central tendency. The median (IQR) duration of hospital stays (for all causes) has remained generally stable at 3 days, but increased to 4 (2-6) days in 2023 (**Figure 3**).

Medians and IQRs of hospitalization durations varied substantially by major diagnostic category. The shortest median durations of hospital stays, at 2 (2-6) days were observed for disorders of the musculoskeletal, genitourinary, and digestive systems, while the longest were for conditions in the ‘other’ and mental health diagnostic categories, where median (IQR) values were 5 (2-15) and 6 (4-11) days, respectively. Infectious and parasitic diseases had a median of 4 (2-6) days, and the remaining categories had a median of 3 (2-6 days).

**Figure 4:**
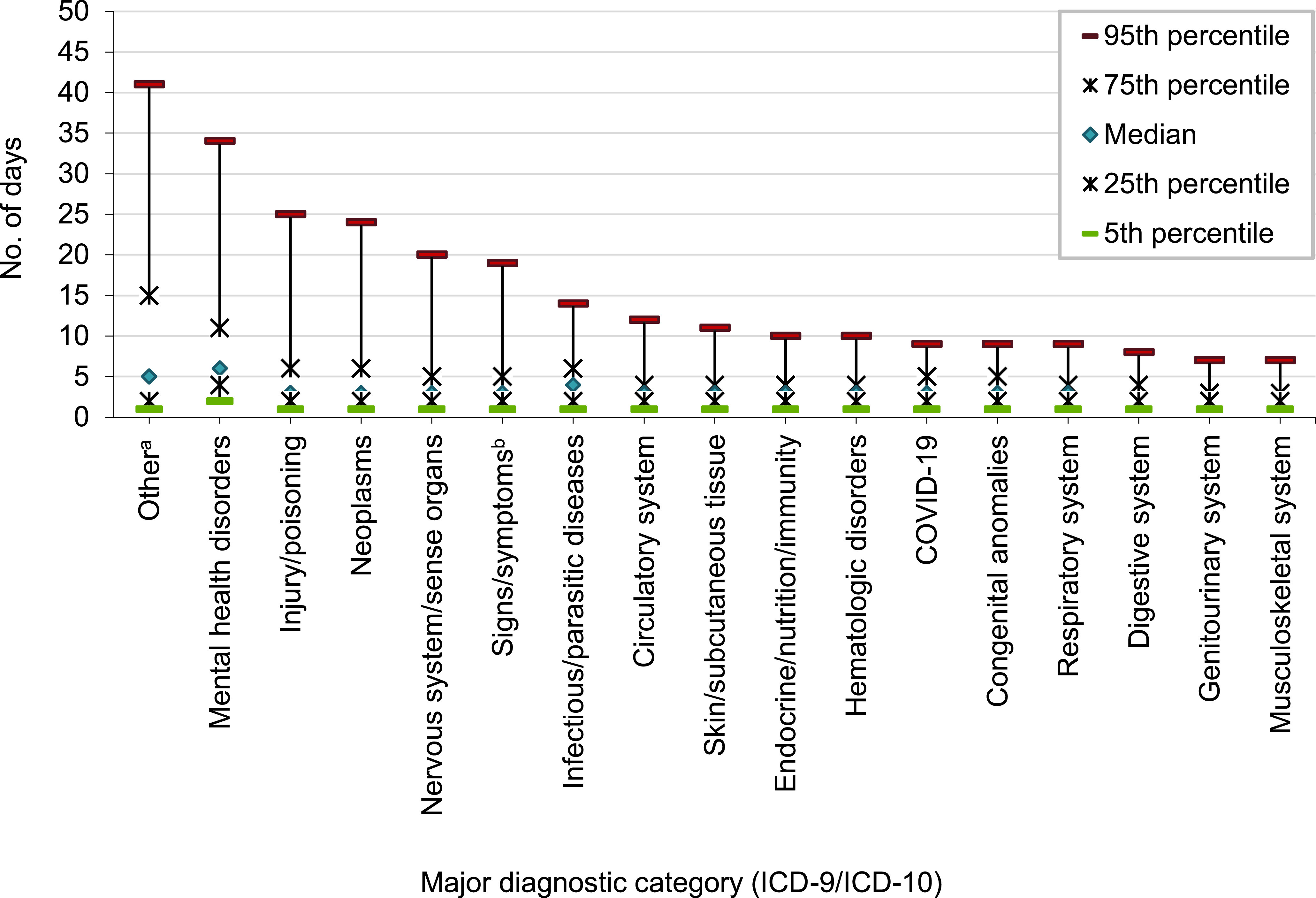
Length of Hospital Stay by ICD-10 Major Diagnostic Category, Active Component, U.S. Armed Forces, 2023

Five percent of hospitalization stays exceeded 10 days for one half of ICD diagnostic categories: skin and subcutaneous tissue (11 days), circulatory system disorders (12 days), signs, symptoms and ill-defined conditions (19 days), nervous system/sense organ disorders (20 days), neoplasms (24 days), injury/poisoning (25 days), mental health disorders (34 days), and other non-pregnancy-related factors influencing health status and contact with health services (primarily orthopedic aftercare and rehabilitation following prior illness or injury) (41 days) (**Figure 4**).

**Hospitalizations, by Service**

Among active component members of the Air Force and Space Force, pregnancy- and delivery-related conditions accounted for more hospitalizations than any other illnesses or injury category, while among active component members of the Army, Navy, and Marine Corps, mental health disorders were the leading cause of hospitalization (**Table 4**). This pattern has been observed in recent years. Prior to 2020, pregnancy- and delivery-related conditions were ranked first for both Navy and Air Force active component members. Among all the services, the crude hospitalization rate for mental health disorders was highest among active component Army members (17.7 per 1,000 p-yrs).

Injury was the third leading hospitalization category for all services, except the Air Force, where it was ranked fourth. The hospitalization rate for injury was highest among Army (5.3 per 1,000 p-yrs) and Marine Corps members (4.9 per 1,000 p-yrs), and lowest among Air Force and Space Force members (2.7 and 2.2 per 1,000 p-yrs, respectively); this service-ranked distribution has been observed since 2010.

## 2 DISCUSSION

The crude annual hospitalization rate observed in 2023 marks the lowest recorded level from 2014 until 2023. Hospitalization rates demonstrated a general decreasing trend, with annual fluctuations, throughout the reporting period. In 2020 there was a significant decline, coincident with COVID-19-related changes in health care provision.

As in past years, in 2023 mental health disorders, pregnancy- and delivery-related conditions, and injury accounted for more than half of all active component hospitalizations. Adjustment disorders, alcohol dependence, depressive disorders, and PTSD were among the leading primary discharge diagnoses for both men and women. The continued decline of hospitalization frequencies and rates is attributed to a generalized decline among the major diagnostic categories since 2019, with substantial declines in the musculoskeletal system, injury, and ‘other’ categories.

Certain limitations should be considered when interpreting these results. This summary is based on primary (first-listed) discharge diagnoses only, but in many hospitalized cases multiple conditions can be present; for example, joint pain (category: musculoskeletal) may be co-listed with an injury (category: injury). In such cases, only the first-listed discharge diagnosis would be accounted in this report. This could underestimate hospitalization rates for common conditions by dividing them among 2 or more subcategories.

This is the second year DMSS data were housed and analyzed from the Military Health System Information Platform (MIP). Additionally, nearly all military treatment facilities are using GENESIS software to electronically capture medical care. Data completeness issues with data transfers between GENESIS to the Medical Data Store (MDR) to DMSS have improved significantly since last year’s report. Regardless of whatever electronic system is used to capture hospitalization information, every hospitalization record requires completion of a discharge summary before the event record is reported in the system. Consequently, timeliness of reporting can still be an issue that may lead to underestimates of true counts and rates of hospitalizations for the most recent year of reporting. As a result, direct comparison between the 2023 data and data from prior years should be interpreted with caution.

## Figures and Tables

**Table 1:** Numbers, Rates^a^, and Ranks^b^ of Hospitalizations by ICD-10 Major Diagnostic Category, Active Component, U.S. Armed Forces, 2019, 2021, and 2023

	2019			2021			2023		
Major Diagnostic Category (ICD-10)	No.	Rate	Rank	No.	Rate	Rank	No.	Rate	Rank
Mental disorders (F01-F99)	19,845	15.1	1	21,732	16.29	1	19,534	15.2	1
Pregnancy and delivery (O00-O9A, relevant Z codes)^c^	16,277	73.5	2	17,596	76.10	2	16,085	70.9	2
Injury and poisoning (S00-T88, DOD0101-DOD0105)	6,674	5.1	3	6,023	4.51	3	5,445	4.2	3
Digestive system (K00-K95)	5,627	4.3	4	5,427	4.07	4	4,798	3.7	4
Musculoskeletal system (M00-M99)	4,924	3.7	5	4,016	3.01	5	3,651	2.8	5
Signs, symptoms, and ill-defined conditions (R00-R99)	3,134	2.4	6	2,767	2.07	6	2,274	1.8	6
Circulatory system (I00-I99)	1,762	1.3	10	1,700	1.27	9	1,659	1.3	7
Genitourinary system (N00-N99)	1,989	1.5	9	1,780	1.33	7	1,477	1.1	8
Respiratory system (J00-J99, U07.0)	2,188	1.7	8	1,268	0.95	12	1,391	1.1	9
Nervous system and sense organs (G00-G99, H00-H95)	1,592	1.2	11	1,342	1.01	10	1,303	1.0	10
Other (Z00–Z99, except pregnancy-related)^d^	2,269	1.7	7	1,764	1.32	8	1,146	0.9	11
Neoplasms (C00-D49)	1,422	1.1	12	1,315	0.99	11	1,135	0.9	12
Infectious and parasitic diseases (A00-B99)	1,137	0.9	13	968	0.73	14	1,006	0.8	13
Skin and subcutaneous tissue (L00-L99)	1,107	0.8	14	743	0.56	15	769	0.6	14
Endocrine, nutrition, immunity (E00-E89)	554	0.4	15	569	0.43	16	540	0.4	15
Hematologic and immune disorders (D50-D89)	303	0.2	16	310	0.23	17	267	0.2	16
Congenital anomalies (Q00-Q99)	261	0.2	17	241	0.18	18	243	0.2	17
COVID-19 (U07.1, U09.9)	--	--	--	1,063	0.80	13	83	0.1	18
Total	71,065	54.0		70,624	52.93		62,806	48.8	

Abbreviations: ICD-10, International Classification of Diseases, 10th Revision; No., number; COVID-19, coronavirus disease 2019.

^a^ Rate per 1,000 person-years.

^b^ Rank of major diagnostic category based on number of hospitalizations.

^c^ Rate of pregnancy and delivery-related hospitalizations among females only.

^d^ Other factors influencing health status and contact with health services (excluding pregnancy-related).

**Table 2:** Numbers and Percentages of the Most Frequent Diagnoses During Hospitalization Among Men by ICD-10 Major Diagnostic Category, Active Component, U.S. Armed Forces, 2023

Diagnostic category (ICD-10 codes)	No.	%^a^
**Mental health disorders (F01-F99)**	**14,949**	
Adjustment disorders	4,927	33.0
Alcohol dependence	2,696	18.0
Major depressive disorder, recurrent severe without psychotic features	1,480	9.9
Post-traumatic stress disorder (PTSD)	623	4.2
Alcohol abuse	522	3.5
**Injury and poisoning (S00–T98, DOD0101–DOD0105)**	**4,629**	
Infection following a procedure	203	4.4
Concussion	176	3.8
Other fractures of lower leg	139	3.0
Fracture of shaft of tibia	132	2.9
Unspecified injury	114	2.5
**Digestive system (K00–K95)**	**3,828**	
Other and unspecified acute appendicitis	901	23.5
Acute appendicitis with localized peritonitis	252	6.6
Acute pancreatitis, unspecified	162	4.2
Alcohol induced acute pancreatitis	139	3.6
Other and unspecified intestinal obstruction	109	2.8
**Musculoskeletal system (M00–M99)**	**2,990**	
Other specified disorders of muscle	510	17.1
Thoracic, thoracolumbar and lumbosacral intervertebral disc disorders with radiculopathy	239	8.0
Spinal stenosis	202	6.8
Major anomalies of jaw size	137	4.6
Anomalies of dental arch relationship	124	4.1
**Symptoms, signs and abnormal clinical and laboratory findings, NEC (R00–R99)**	**1,723**	
Other symptoms and signs involving emotional state	398	23.1
Syncope and collapse	125	7.3
Other symptoms and signs involving cognitive functions and awareness	119	6.9
Unspecified abdominal pain	100	5.8
Chest pain, unspecified	99	5.7
**Circulatory system (I00–I99)**	**1,439**	
Pulmonary embolism without acute cor pulmonale	137	9.5
Non-ST elevation (NSTEMI) myocardial infarction	91	6.3
Paroxysmal atrial fibrillation	75	5.2
Unspecified atrial fibrillation and atrial flutter	42	2.9
Atherosclerotic heart disease of native coronary artery	40	2.8
**Respiratory system (J00-J99, U07.0)**	**1,172**	
Pneumonia, unspecified organism	134	11.4
Peritonsillar abscess	113	9.6
Deviated nasal septum	92	7.8
Other and unspecified asthma	63	5.4
Other pneumothorax and air leak	59	5.0
**Nervous system and sense organs (G00–G99, H00–H95)**	**1,003**	
Sleep apnea	86	8.6
Epilepsy, unspecified	54	5.4
Acute pain, not elsewhere classified	39	3.9
Brachial plexus disorders	36	3.6
Migraine with aura	30	3.0
**Other (Z00–Z99, except pregnancy-related)^b^**	**888**	
Encounter for antineoplastic chemotherapy and immunotherapy	161	18.1
Aftercare following joint replacement surgery	108	12.2
Encounter for examination and observation for unspecified reason	99	11.1
Encounter for other orthopedic aftercare	88	9.9
Encounter for other specified postprocedural aftercare	68	7.7
**Infectious and parasitic diseases (A00–B99)**	**765**	
Sepsis, unspecified organism	315	41.2
Infectious gastroenteritis and colitis, unspecified	54	7.1
Other specified sepsis	30	3.9
Infectious mononucleosis, unspecified	25	3.3
Enterocolitis due to *Clostridioides difficile*	21	2.7
**Genitourinary system (N00–N99)**	**731**	
Acute kidney failure, unspecified	170	23.3
Hydronephrosis with renal and ureteral calculous obstruction	82	11.2
Calculus of kidney	43	5.9
Calculus of ureter	34	4.7
Urethral stricture, unspecified	33	4.5
**Neoplasms (C00–D49)**	**723**	
Malignant neoplasm of brain, unspecified	38	5.3
Malignant neoplasm of thyroid gland	37	5.1
Benign neoplasm of pituitary gland	22	3.0
Acute myeloblastic leukemia	21	2.9
Malignant neoplasm of frontal lobe	18	2.5
**Skin and subcutaneous tissue (L00–L99)**	**665**	
Cellulitis and acute lymphangitis of other parts of limb	286	43.0
Cellulitis and acute lymphangitis of face and neck	45	6.8
Cellulitis and acute lymphangitis of finger and toe	34	5.1
Cutaneous abscess, furuncle and carbuncle of limb	33	5.0
Cutaneous abscess, furuncle and carbuncle of trunk	26	3.9
**Endocrine, nutrition, immunity (E00–E89)**	**420**	
Type 2 diabetes mellitus with ketoacidosis	57	13.6
Type 2 diabetes mellitus with other specified complications	50	11.9
Type 1 diabetes mellitus with ketoacidosis	43	10.2
Dehydration	34	8.1
Hypo-osmolality and hyponatremia	29	6.9
**Hematologic and immune disorders (D50–D89)**	**181**	
Neutropenia, unspecified	26	14.4
Acute posthemorrhagic anemia	19	10.5
Other specified aplastic anemias and other bone marrow failure syndromes	17	9.4
Iron deficiency anemia, unspecified	16	8.8
Immune thrombocytopenic purpura	13	7.2
**Congenital anomalies (Q00–Q99)**	**151**	
Atrial septal defect	19	12.6
Malformation of coronary vessels	14	9.3
Other congenital deformities of hip	13	8.6
Arteriovenous malformation of cerebral vessels	11	7.3
Meckel's diverticulum (displaced) (hypertrophic)	10	6.6
**COVID-19 (ICD-10: U07.1, U09.9)**	**68**	
COVID-19	67	98.5
Post COVID-19 condition, unspecified	1	1.5

Abbreviations: ICD, International Classification of Diseases, 10th Revision; No., number; NSTEMI, non-ST segment elevation myocardial infarction; NEC, not elsewhere classified.

^a^ Percentage of the total number of hospitalizations within the diagnostic category.

^b^ Other factors influencing health status and contact with health services (excluding pregnancy-related).

**Table 3:** Numbers and Percentages of the Most Frequent Diagnoses During Hospitalization Among Women by ICD-10 Major Diagnostic Category, Active Component, U.S. Armed Forces, 2023

Diagnostic category (ICD-10 codes)	No.	%^a^
**Pregnancy and delivery (O00-O99, relevant Z codes)**	**16,085**	
Post-term pregnancy	1,621	10.1
Abnormality in fetal heart rate and rhythm complicating labor and delivery	999	6.2
Maternal care due to uterine scar from previous surgery	987	6.1
Premature rupture of membranes, onset of labor within 24 hours of rupture	860	5.3
Gestational [pregnancy-induced] hypertension without significant proteinuria, complicating childbirth	787	4.9
**Mental health disorders (F01-F99)**	**4,585**	
Adjustment disorders	1,294	28.2
Major depressive disorder, recurrent severe without psychotic features	561	12.2
Post-traumatic stress disorder (PTSD)	495	10.8
Alcohol dependence	295	6.4
Depression, unspecified	157	3.4
**Digestive system (K00–K95)**	**970**	
Other and unspecified acute appendicitis	179	18.5
Calculus of gallbladder with acute cholecystitis	60	6.2
Noninfective gastroenteritis and colitis, unspecified	44	4.5
Acute appendicitis with localized peritonitis	39	4.0
Calculus of gallbladder and bile duct with cholecystitis	31	3.2
**Injury and poisoning (S00–T98, DOD0101–DOD0105)**	**816**	
Poisoning by, adverse effect of and underdosing of 4-Aminophenol derivatives	47	5.8
Poisoning by, adverse effect of and underdosing of other and unspecified antidepressants	43	5.3
Infection following a procedure	38	4.7
Unspecified injury	28	3.4
Fracture of shaft of femur	20	2.5
**Genitourinary system (N00–N99)**	**746**	
Abnormal uterine and vaginal bleeding, unspecified	99	13.3
Other and unspecified ovarian cysts	66	8.8
Hypertrophy of breast	52	7.0
Acute pyelonephritis	33	4.4
Excessive and frequent menstruation with regular cycle	33	4.4
**Musculoskeletal system (M00–M99)**	**661**	
Major anomalies of jaw size	53	8.0
Other specified disorders of muscle	48	7.3
Anomalies of dental arch relationship	44	6.7
Pain in joint	34	5.1
Spinal stenosis	31	4.7
**Symptoms, signs and abnormal clinical and laboratory findings, NEC (R00–R99)**	**551**	
Other symptoms and signs involving emotional state	120	21.8
Unspecified abdominal pain	53	9.6
Syncope and collapse	46	8.3
Pain localized to other parts of lower abdomen	34	6.2
Unspecified convulsions	29	5.3
**Neoplasms (C00–D49)**	**412**	
Leiomyoma of uterus, unspecified	94	22.8
Intramural leiomyoma of uterus	46	11.2
Subserosal leiomyoma of uterus	36	8.7
Malignant neoplasm of thyroid gland	19	4.6
Malignant neoplasm of breast of unspecified site	15	3.6
**Nervous system and sense organs (G00–G99, H00–H95)**	**300**	
Migraine with aura	20	6.7
Multiple sclerosis	18	6.0
Acute pain, not elsewhere classified	16	5.3
Brachial plexus disorders	15	5.0
Epilepsy, unspecified	14	4.7
**Other (Z00–Z99, except pregnancy-related)^b^**	**258**	
Encounter for examination and observation for unspecified reason	49	19.0
Aftercare following joint replacement surgery	35	13.6
Encounter for other specified postprocedural aftercare	25	9.7
Encounter for other orthopedic aftercare	24	9.3
Encounter for prophylactic surgery for risk factors related to malignant neoplasms	19	7.4
**Infectious and parasitic diseases (A00–B99)**	**241**	
Sepsis, unspecified organism	111	46.1
Infectious gastroenteritis and colitis, unspecified	26	10.8
Sepsis due to other Gram-negative organisms	11	4.6
Other specified sepsis	9	3.7
Viral intestinal infection, unspecified	8	3.3
**Circulatory system (I00–I99)**	**220**	
Pulmonary embolism without acute cor pulmonale	31	14.1
Supraventricular tachycardia	10	4.5
Cerebral aneurysm, nonruptured	10	4.5
Non-ST elevation (NSTEMI) myocardial infarction	9	4.1
Other arterial dissection	9	4.1
**Respiratory system (J00-J99, U07.0)**	**219**	
Peritonsillar abscess	21	9.5
Deviated nasal septum	18	8.2
Chronic tonsillitis and adenoiditis	15	6.8
Other intraoperative and postprocedural complications and disorders of respiratory system, not elsewhere classified	14	6.4
Acute respiratory failure	12	5.5
**Endocrine, nutrition, immunity (E00–E89)**	**120**	
Thyrotoxicosis with diffuse goiter	18	15.0
Thyrotoxicosis, unspecified	10	8.3
Dehydration	10	8.3
Type 2 diabetes mellitus with other specified complications	7	5.8
Hypokalemia	6	5.0
**Skin and subcutaneous tissue (L00–L99)**	**104**	
Cellulitis and acute lymphangitis of other parts of limb	25	24.0
Pilonidal cyst and sinus with abscess	13	12.5
Postprocedural hematoma and seroma of skin and subcutaneous tissue following a procedure	9	8.7
Cutaneous abscess, furuncle and carbuncle of limb	8	7.7
Cellulitis and acute lymphangitis of face and neck	8	7.7
**Congenital anomalies (Q00–Q99)**	**92**	
Other congenital deformities of hip	53	57.6
Atrial septal defect	7	7.6
Malformation of coronary vessels	4	4.3
Partial anomalous pulmonary venous connection	2	2.2
Other malformations of cerebral vessels	2	2.2
**Hematologic and immune disorders (D50–D89)**	**86**	
Anemia, unspecified	19	22.1
Iron deficiency anemia, unspecified	14	16.3
Iron deficiency anemia secondary to blood loss (chronic)	10	11.6
Other iron deficiency anemias	7	8.1
Acute posthemorrhagic anemia	7	8.1
**COVID-19 (ICD-10: U07.1, U09.9)**	**15**	
COVID-19	15	100.00

Abbreviations: ICD, International Classification of Diseases, 10th Revision; No., number; NSTEMI, non-ST segment elevation myocardial infarction; NEC, not elsewhere classified.

^a^ Percentage of the total number of hospitalizations within the diagnostic category.

^b^ Other factors influencing health status and contact with health services (excluding pregnancy-related).

**Table 4:** Numbers and Rates^a^ of Hospitalizations, by Service and ICD-10 Diagnostic Category, Active Component, U.S. Armed Forces, 2023

	Army		Navy		Air Force		Space Force		Marine Corps	
Major Diagnostic Category (ICD-10)	No.	Rate	No.	Rate	No.	Rate	No.	Rate	No.	Rate
Mental disorders (F01-F99)	8,069	17.7	5,257	15.8	3,554	11.2	61	7.1	2,593	15.0
Pregnancy and delivery (O00-O9A, relevant Z codes)^b^	5,998	84.2	4,544	65.8	4,249	62.1	87	53.0	1,207	73.3
Injury and poisoning (S00-T88, DOD0101-DOD0105)	2,409	5.3	1,319	4.0	857	2.7	19	2.2	841	4.9
Digestive system (K00-K95)	1,969	4.3	1,299	3.9	960	3.0	16	1.9	554	3.2
Musculoskeletal system (M00-M99)	1,745	3.8	781	2.3	665	2.1	14	1.6	446	2.6
Signs, symptoms, and ill-defined conditions (R00-R99)	1,141	2.5	527	1.6	428	1.3	8	0.9	170	1.0
Circulatory system (I00-I99)	685	1.5	468	1.4	353	1.1	8	0.9	145	0.8
Genitourinary system (N00-N99)	648	1.4	328	1.0	360	1.1	9	1.0	132	0.8
Respiratory system (J00-J99, U07.0)	622	1.4	309	0.9	234	0.7	4	0.5	222	1.3
Nervous system and sense organs (G00-G99, H00-H95)	575	1.3	326	1.0	276	0.9	5	0.6	121	0.7
Other (Z00–Z99, except pregnancy-related)^c^	451	1.0	249	0.7	266	0.8	6	0.7	174	1.0
Neoplasms (C00-D49)	437	1.0	338	1.0	254	0.8	8	0.9	98	0.6
Infectious and parasitic diseases (A00-B99)	361	0.8	272	0.8	237	0.7	4	0.5	132	0.8
Skin and subcutaneous tissue (L00-L99)	339	0.7	153	0.5	117	0.4	3	0.3	157	0.9
Endocrine, nutrition, immunity (E00-E89)	207	0.5	154	0.5	123	0.4	4	0.5	52	0.3
Hematologic and immune disorders (D50-D89)	116	0.3	54	0.2	48	0.2	3	0.3	46	0.3
Congenital anomalies (Q00-Q99)	103	0.2	64	0.2	47	0.1	1	0.1	28	0.2
COVID-19 (U07.1, U09.9)	44	0.1	15	0.0	13	0.0			11	0.1
Total	25,919	57.0	16,457	49.5	13,041	41.0	260	30.2	7,129	41.3

Abbreviations: ICD-10, International Classification of Diseases, 10th Revision; No., number; COVID-19, coronavirus disease 2019.

^a^ Rates are based on 1,000 person-years.

^b^ Rates for pregnancy and delivery-related hospitalizations among females only (in parentheses).

^c^ Other factors influencing health status and contact with health services (excluding pregnancy-related).
